# The complete mitochondrial genome of *Melanoides tuberculata* (Müller, 1774) in Guangdong, China

**DOI:** 10.1080/23802359.2022.2054735

**Published:** 2022-07-22

**Authors:** Yu Ling, Yanru Zhang, Jacob Njaramba Ngatia, Huanmin Zhou

**Affiliations:** a College of Life Science, Inner Mongolia Agricultural University, Hohhot, China; b College of Wildlife Resources, Northeast Forestry University, Harbin, China

**Keywords:** *Melanoides tuberculata*, phylogenetic tree, mitochondrial genome

## Abstract

The *Melanoides tuberculata* is an invasive species, which is natively distributed in Africa and Southeast Asia. This study describes the first mitochondrial genome of the *M. tuberculata* based on the whole genome sequencing data. The complete sequence length of the mitogenome is 15,821 bp, including 37 genes (2 rRNA genes, 22 tRNA genes and 13 protein-coding genes). Phylogenetic analysis using the 13 species of Cerithioidea species showed that the *M. tuberculata* is closely related to *P. dartevellei*, forming the sister group to *C. sinensis* and *C. obtuse*.

*Melanoides tuberculata* is commonly known as the red-rimmed melania. This is a small freshwater snail that belongs to the family of Thiaridae (Caenogastropoda: Cerithioidea). This snail is considered as an invasive species, which is natively distributed in Africa, Southeast Asia, China, Malaysia and India. Presently, this species is distributed all over the world (Quirós-Rodríguez et al. 2018). *M. tuberculate* is a potential middle host of several parasites, which can transmit diseases between human and other animals (Pointier et al. 1998; Pinto and Melo 2010). The genetic basis of the invasiveness of the *M. tuberculate* is of vital importance for the protection of local ecological systems and the public health.

Mitochondrial DNA is particularly suitable for the study of evolutionary relationships due to its unique characteristics such as high copy number, maternal inheritance and high evolutionary rate (Stoneking and Soodyall 1996). The present study reports the first complete mitochondrial genome of the *M. tuberculate,* as well as the phylogenetic relationships within the superfamily of Cerithioidea. This will provide valuable genetic data for future studies on the *M. tuberculate* and its related species.

The genomic DNA was isolated from the dissected abdominal foot of one individual collected from Guangzhou, Guangdong province, China (latitude: 113.4164 and longitude: 23.0968). This specimen and corresponding genomic DNA were deposited at Inner Mongolia University (https://www.imu.edu.cn/, Yu Ling, lingyuolcuc@gmail.com) under the voucher number SSDCF20150503 and SSDCFGDNA20210421, respectively. The genomic DNA was then sheared into small fragments for preparation of one paired-end genomic DNA library, which was used for whole genome sequencing (WGS). Approximately, 12 Mb raw reads were generated and used for mitochondrial genome assembly. The assembly of the complete mitochondrial genome was performed using the NOVOPlasty version 4.3 (Dierckxsens et al. 2017). MITOS2 (http://mitos2.bioinf.uni-leipzig.de/) was used for final gene annotation and corrections were done manually based on the annotations of its closely related species.

The total length of our assembled mitochondrial genome was 15,821 bp, which comprised of 34.92％ of Adenine (A), 16.33％ of Cytosine (C), 17.32％ of Guanine (G) and 31.43％ of Thiamin (T). Similar to other Cerithioidea species, there also exists a significant AT bias (66.35％). This mitochondrial genome included two ribosomal RNA (rRNA), 22 transfer RNA (tRNA) genes and 13 protein-coding genes (PCGs). The total length of the 13 PCGs was 11,307 bp. The start codon of all 13 PCGs was ATG. However, there were two types of termination codons, including TAG for *nad4l* and *nad2* and TAA for the other 11 PCGs. Similar gene organization and composition between Cerithioidea species have shown the high conservation in the evolution of mitochondrial genome (Figure 1).

**Figure 1.**
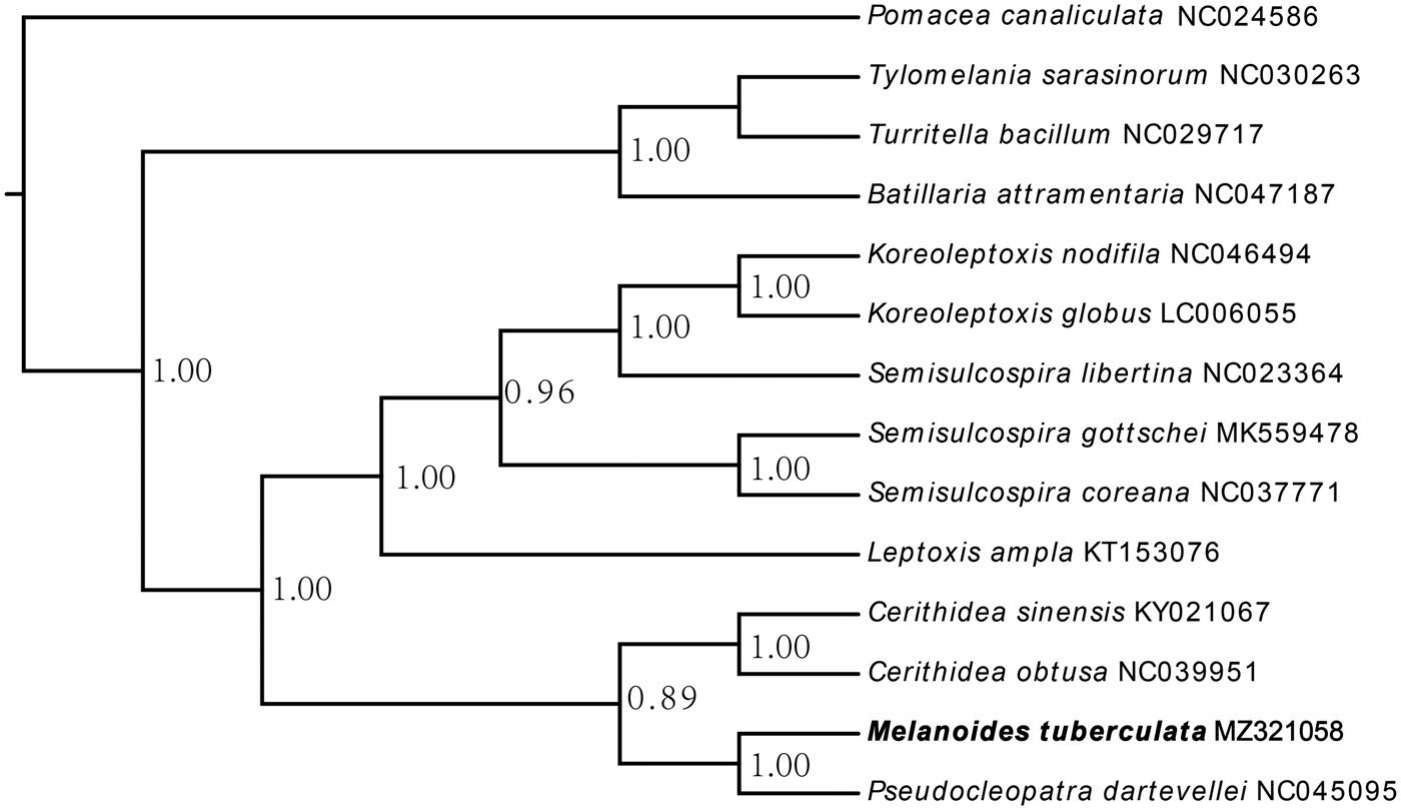
The phylogenetic tree inferred by the complete mitogenomes of 13 Cerithioidea species.

Twelve more mitochondrial genome sequences of snails from the superfamily Cerithioidea were downloaded to further explore the phylogenetic relationships with *M. tuberculate*. We selected the *Pomacea canaliculata* as the outgroup. Thirteen PCGs from these fourteen species were used for phylogenetic tree inference. GTR was determined as the best substitution model using the Akaike Information Criterion (AIC) as implemented in jModeltest 2.1.3 (Darriba et al. 2012). Phylogenetic tree was constructed using the MrBayes v3.2 (Ronquist et al. 2012) with *ngen = 100,000* and *samplefreq = 100* parameters. This phylogenetic tree showed two distinct clusters in the superfamily Cerithioidea, and the *M. tuberculata* was a sister group to *P. dartevellei*, and formed the sister cluster to *C. sinensis* and *C. obtuse*, with high posterior probability (1.00).

## Data Availability

The complete mitochondrial genome sequences generated in this study is openly available in NCBI website (http://www.ncbi.nlm.nih.gov) under the accession number of MZ321058. The associated BioProject, SRA, and Bio-Sample numbers are PRJNA737044, SRP323795, and SAMN19678505, respectively.
